# Diethyl 4-(4-cyano­phen­yl)-2,6-dimethyl-1,4-dihydro­pyridine-3,5-dicarboxyl­ate

**DOI:** 10.1107/S1600536810018155

**Published:** 2010-05-22

**Authors:** Peng Zhang, Weiqun Zhu

**Affiliations:** a School of Chemistry and Chemical Engineering, Shandong University, Jinan 250100, People’s Republic of China

## Abstract

In the title compound, C_20_H_22_N_2_O_4_, the dihedral angle between the roughly planar dihydro­pyridine ring (r.m.s. deviation = 0.092 Å) and the benzene ring is 87.09 (6)°. One of the eth­oxy side chains is disordered over two orientations in a 0.669 (14):0.331 (14) ratio. In the crystal, mol­ecules are linked by N—H⋯N hydrogen bonds, generating chains.

## Related literature

For general background to dihydro­pyridine derivatives, see: Gaudio *et al.* (1994 ▶). 
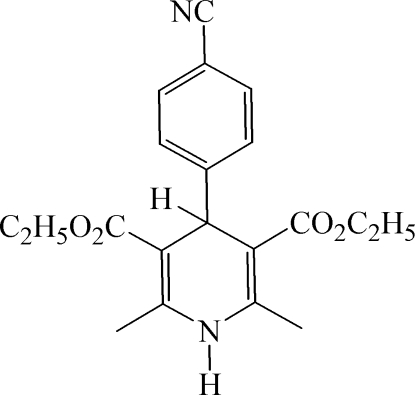


## Experimental

### 

#### Crystal data

C_20_H_22_N_2_O_4_
*M* *_r_* = 354.40
Monoclinic, 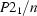

*a* = 10.4596 (13) Å
*b* = 9.5117 (12) Å
*c* = 19.160 (2) Å
β = 91.493 (1)°
*V* = 1905.6 (4) Å^3^
*Z* = 4
Mo *K*α radiation
μ = 0.09 mm^−1^
*T* = 296 K
0.12 × 0.10 × 0.08 mm

#### Data collection

Bruker APEXII CCD diffractometer
Absorption correction: multi-scan (*SADABS*; Bruker, 2001 ▶) *T*_min_ = 0.990, *T*_max_ = 0.993
10000 measured reflections
3298 independent reflections
2408 reflections with *I* > 2σ(*I*)
*R*_int_ = 0.020

#### Refinement

*R*[*F* ^2^ > 2σ(*F* ^2^)] = 0.051
*wR*(*F* ^2^) = 0.162
*S* = 1.02
3298 reflections
249 parameters
2 restraints
H-atom parameters constrained
Δρ_max_ = 0.31 e Å^−3^
Δρ_min_ = −0.28 e Å^−3^

### 

Data collection: *APEX2* (Bruker, 2004 ▶); cell refinement: *SAINT-Plus* (Bruker, 2001 ▶); data reduction: *SAINT-Plus*; program(s) used to solve structure: *SHELXS97* (Sheldrick, 2008 ▶); program(s) used to refine structure: *SHELXL97* (Sheldrick, 2008 ▶); molecular graphics: *SHELXTL* (Sheldrick, 2008 ▶); software used to prepare material for publication: *SHELXTL*.

## Supplementary Material

Crystal structure: contains datablocks global, I. DOI: 10.1107/S1600536810018155/hb5444sup1.cif

Structure factors: contains datablocks I. DOI: 10.1107/S1600536810018155/hb5444Isup2.hkl

Additional supplementary materials:  crystallographic information; 3D view; checkCIF report

## Figures and Tables

**Table 1:** Hydrogen-bond geometry (Å, °)

*D*—H⋯*A*	*D*—H	H⋯*A*	*D*⋯*A*	*D*—H⋯*A*
N2—H2⋯N1^i^	0.86	2.32	3.098 (3)	150

Symmetry code: (i) 
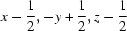
.

**Fig. 1.**
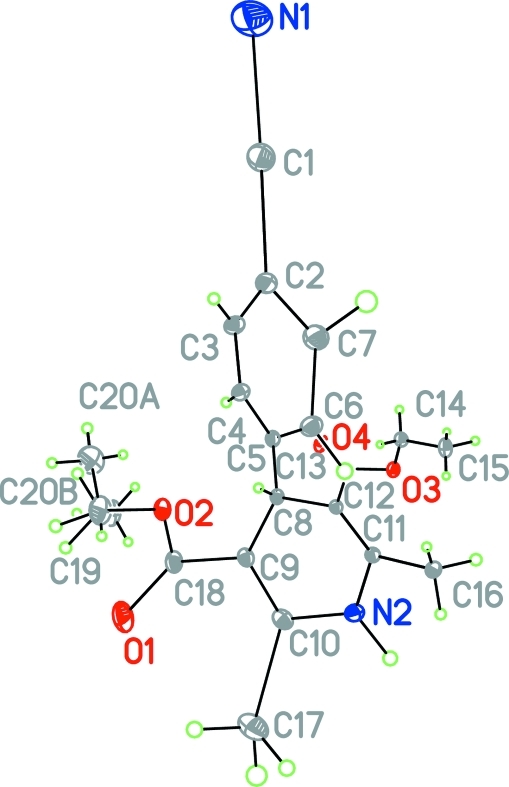
The molecular structure of (I) showing displacement ellipsoids drawn at the 30% probability level.

**Fig. 2.**
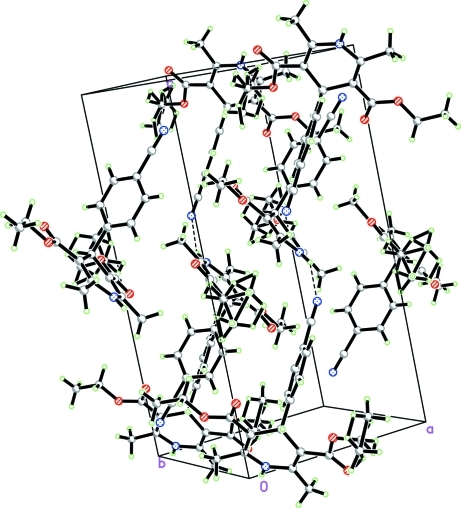
The crystal packing of (I), displayed with N—H···N hydrogen bonds as dashed lines.

## supplementary crystallographic information

### Comment

The synthesis of 1,4-dihydropyridine derivatives has attracted continuous research interest due to various vasodilator, anti-hypertensive, bronchodilator, heptaprotective, anti-tumor, anti-mutagenic, geroprotective and anti-diabetic agents (Gaudio *et al.*, 1994). Here, we describe the recystallization and structural characterization of the title compound.

The molecular structure is shown in Fig 1. The dihedral angle between the two rings is 87.09 (6) °. The mean devation of the dihydropyridine plane is 0.0824 Å. The intermolecular hydrogen bonding of N2—H2···N1 leads to a consolidation of the structure (Fig. 2; Table 1).

### Experimental

Diethyl 2,6-dimethyl-4-(4-cyanophenyl)-1,4-dihydropyridine-3,5-dicarboxylate (1 mmol 0.39 g) was dissolved in 20 ml ethanol was evaporated in one open flask at room temperature. One week later, yellow blocks of (I) were obained. Anal. C_20_H_22_N_2_O_4_: C, 67.72; H, 5.64; N, 7.90 %. Found: C, 67.56; H, 5.46; N, 7.61 %.

### Refinement

All hydrogen atoms bound to aromatic carbon atoms were refined in calculated positions using a riding model with a C—H distance of 0.93 Å and *U*_iso_ = 1.2*U*_eq_(C). Hydrogen atoms attached to aromatic N atoms were refined with a N—H distance of 0.86 Å and *U*_iso_ = 1.2*U*_eq_(N).

### Crystal data

**Table:**

C_20_H_22_N_2_O_4_	*F*(000) = 752
*M**_r_* = 354.40	*D*_x_ = 1.235 Mg m^−^^3^
Monoclinic, *P*2_1_/*n*	Mo *K*α radiation, λ = 0.71073 Å
Hall symbol: -P 2yn	Cell parameters from 3532 reflections
*a* = 10.4596 (13) Å	θ = 2.4–25.9°
*b* = 9.5117 (12) Å	µ = 0.09 mm^−^^1^
*c* = 19.160 (2) Å	*T* = 296 K
β = 91.493 (1)°	Block, colorless
*V* = 1905.6 (4) Å^3^	0.12 × 0.10 × 0.08 mm
*Z* = 4	

### Data collection

**Table:**

Bruker APEXII CCD diffractometer	3298 independent reflections
Radiation source: fine-focus sealed tube	2408 reflections with *I* > 2σ(*I*)
graphite	*R*_int_ = 0.020
phi and ω scans	θ_max_ = 25.0°, θ_min_ = 2.2°
Absorption correction: multi-scan (*SADABS*; Bruker, 2001)	*h* = −12→10
*T*_min_ = 0.990, *T*_max_ = 0.993	*k* = −9→11
10000 measured reflections	*l* = −22→22

### Refinement

**Table:**

Refinement on *F*^2^	Secondary atom site location: difference Fourier map
Least-squares matrix: full	Hydrogen site location: inferred from neighbouring sites
*R*[*F*^2^ > 2σ(*F*^2^)] = 0.051	H-atom parameters constrained
*wR*(*F*^2^) = 0.162	*w* = 1/[σ^2^(*F*_o_^2^) + (0.0857*P*)^2^ + 0.6251*P*] where *P* = (*F*_o_^2^ + 2*F*_c_^2^)/3
*S* = 1.02	(Δ/σ)_max_ = 0.001
3298 reflections	Δρ_max_ = 0.31 e Å^−^^3^
249 parameters	Δρ_min_ = −0.28 e Å^−^^3^
2 restraints	Extinction correction: *SHELXL97* (Sheldrick, 2008), Fc^*^=kFc[1+0.001xFc^2^λ^3^/sin(2θ)]^-1/4^
Primary atom site location: structure-invariant direct methods	Extinction coefficient: 0.012 (2)

### Special details

**Table:**

Geometry. All esds (except the esd in the dihedral angle between two l.s. planes) are estimated using the full covariance matrix. The cell esds are taken into account individually in the estimation of esds in distances, angles and torsion angles; correlations between esds in cell parameters are only used when they are defined by crystal symmetry. An approximate (isotropic) treatment of cell esds is used for estimating esds involving l.s. planes.
Refinement. Refinement of *F*^2^ against ALL reflections. The weighted *R*-factor wR and goodness of fit *S* are based on *F*^2^, conventional *R*-factors *R* are based on *F*, with *F* set to zero for negative *F*^2^. The threshold expression of *F*^2^ > σ(*F*^2^) is used only for calculating *R*-factors(gt) etc. and is not relevant to the choice of reflections for refinement. *R*-factors based on *F*^2^ are statistically about twice as large as those based on *F*, and *R*- factors based on ALL data will be even larger.

### Fractional atomic coordinates and isotropic or equivalent isotropic displacement parameters (Å^2^)

**Table:**

	*x*	*y*	*z*	*U*_iso_*/*U*_eq_	Occ. (<1)
C1	0.5366 (2)	0.1062 (3)	0.32734 (12)	0.0574 (6)	
C2	0.4507 (2)	0.0690 (2)	0.27004 (10)	0.0483 (5)	
C3	0.3890 (2)	−0.0596 (3)	0.26959 (12)	0.0607 (6)	
H3	0.4024	−0.1222	0.3063	0.073*	
C4	0.3078 (2)	−0.0948 (2)	0.21466 (12)	0.0573 (6)	
H4	0.2670	−0.1817	0.2145	0.069*	
C5	0.28599 (19)	−0.0030 (2)	0.15987 (10)	0.0419 (5)	
C6	0.3497 (2)	0.1239 (2)	0.16056 (12)	0.0535 (6)	
H6	0.3378	0.1854	0.1233	0.064*	
C7	0.4306 (2)	0.1614 (2)	0.21529 (12)	0.0552 (6)	
H7	0.4713	0.2483	0.2154	0.066*	
C8	0.19219 (19)	−0.0393 (2)	0.09975 (11)	0.0444 (5)	
H8	0.1533	−0.1305	0.1098	0.053*	
C9	0.2616 (2)	−0.0512 (2)	0.03111 (12)	0.0507 (6)	
C10	0.2559 (2)	0.0529 (3)	−0.01647 (11)	0.0528 (6)	
C11	0.08375 (19)	0.1693 (2)	0.04492 (10)	0.0445 (5)	
C12	0.08624 (18)	0.0706 (2)	0.09551 (10)	0.0425 (5)	
C13	−0.0033 (2)	0.0607 (2)	0.15302 (12)	0.0490 (5)	
C14	−0.1644 (3)	0.1750 (3)	0.21848 (15)	0.0738 (8)	
H14A	−0.1152	0.1750	0.2621	0.089*	
H14B	−0.2190	0.0925	0.2175	0.089*	
C15	−0.2415 (3)	0.3012 (3)	0.2130 (2)	0.0980 (11)	
H15A	−0.1871	0.3823	0.2168	0.147*	
H15B	−0.3019	0.3025	0.2498	0.147*	
H15C	−0.2864	0.3024	0.1687	0.147*	
C16	−0.0089 (2)	0.2888 (3)	0.03443 (13)	0.0586 (6)	
H16A	−0.0883	0.2659	0.0557	0.088*	
H16B	−0.0235	0.3045	−0.0146	0.088*	
H16C	0.0261	0.3724	0.0555	0.088*	
C17	0.3287 (3)	0.0650 (4)	−0.08253 (13)	0.0756 (8)	
H17A	0.4001	0.1272	−0.0752	0.113*	
H17B	0.2735	0.1017	−0.1190	0.113*	
H17C	0.3592	−0.0261	−0.0958	0.113*	
C18	0.3377 (2)	−0.1776 (3)	0.01887 (15)	0.0676 (7)	
C19	0.3950 (6)	−0.3990 (5)	0.0605 (3)	0.158 (2)	
H19A	0.3515	−0.4556	0.0251	0.190*	0.669 (14)
H19B	0.4793	−0.3765	0.0437	0.190*	0.669 (14)
H19C	0.4473	−0.3909	0.0196	0.190*	0.331 (14)
H19D	0.4514	−0.4147	0.1007	0.190*	0.331 (14)
C20A	0.4079 (13)	−0.4759 (9)	0.1207 (5)	0.185 (6)	0.669 (14)
H20A	0.4549	−0.5603	0.1116	0.278*	0.669 (14)
H20B	0.3248	−0.4999	0.1371	0.278*	0.669 (14)
H20C	0.4530	−0.4213	0.1556	0.278*	0.669 (14)
C20B	0.3114 (12)	−0.5133 (12)	0.0522 (12)	0.141 (9)	0.331 (14)
H20D	0.3600	−0.5980	0.0467	0.212*	0.331 (14)
H20E	0.2573	−0.4988	0.0116	0.212*	0.331 (14)
H20F	0.2598	−0.5213	0.0927	0.212*	0.331 (14)
N1	0.6066 (2)	0.1329 (3)	0.37179 (12)	0.0765 (7)	
N2	0.17490 (18)	0.1647 (2)	−0.00618 (9)	0.0534 (5)	
H2	0.1813	0.2363	−0.0333	0.064*	
O1	0.4059 (2)	−0.2026 (3)	−0.02939 (13)	0.1062 (8)	
O2	0.3246 (2)	−0.2705 (2)	0.06982 (13)	0.0967 (7)	
O3	−0.07949 (16)	0.17231 (17)	0.16008 (9)	0.0641 (5)	
O4	−0.00600 (19)	−0.0369 (2)	0.19268 (11)	0.0824 (6)	

### Atomic displacement parameters (Å^2^)

**Table:**

	*U*^11^	*U*^22^	*U*^33^	*U*^12^	*U*^13^	*U*^23^
C1	0.0631 (14)	0.0625 (15)	0.0467 (13)	0.0055 (12)	−0.0004 (11)	−0.0063 (11)
C2	0.0482 (12)	0.0555 (13)	0.0413 (11)	0.0057 (10)	−0.0013 (9)	−0.0061 (9)
C3	0.0722 (15)	0.0591 (15)	0.0503 (13)	−0.0007 (12)	−0.0094 (12)	0.0147 (11)
C4	0.0642 (14)	0.0456 (13)	0.0617 (14)	−0.0084 (11)	−0.0080 (11)	0.0095 (10)
C5	0.0410 (10)	0.0396 (11)	0.0452 (11)	0.0051 (9)	0.0025 (8)	−0.0017 (8)
C6	0.0648 (14)	0.0462 (13)	0.0489 (12)	−0.0052 (11)	−0.0103 (11)	0.0075 (10)
C7	0.0603 (13)	0.0466 (12)	0.0582 (13)	−0.0064 (11)	−0.0068 (11)	−0.0006 (10)
C8	0.0446 (11)	0.0371 (11)	0.0513 (12)	−0.0006 (9)	−0.0025 (9)	−0.0032 (9)
C9	0.0453 (12)	0.0539 (13)	0.0525 (12)	0.0024 (10)	−0.0052 (10)	−0.0144 (10)
C10	0.0461 (12)	0.0658 (15)	0.0464 (12)	−0.0015 (11)	−0.0022 (9)	−0.0127 (11)
C11	0.0433 (11)	0.0461 (12)	0.0436 (11)	0.0011 (9)	−0.0056 (9)	−0.0029 (9)
C12	0.0398 (11)	0.0418 (11)	0.0456 (11)	−0.0011 (9)	−0.0029 (9)	−0.0042 (9)
C13	0.0465 (12)	0.0448 (12)	0.0557 (12)	−0.0004 (10)	0.0026 (10)	0.0015 (10)
C14	0.0735 (17)	0.0753 (18)	0.0740 (17)	0.0031 (14)	0.0293 (14)	−0.0027 (14)
C15	0.088 (2)	0.074 (2)	0.135 (3)	0.0022 (16)	0.058 (2)	−0.0069 (19)
C16	0.0624 (14)	0.0569 (14)	0.0562 (13)	0.0106 (11)	−0.0044 (11)	0.0070 (11)
C17	0.0687 (16)	0.106 (2)	0.0526 (14)	0.0028 (15)	0.0107 (12)	−0.0091 (14)
C18	0.0628 (15)	0.0681 (17)	0.0715 (17)	0.0159 (13)	−0.0080 (13)	−0.0218 (14)
C19	0.203 (5)	0.090 (3)	0.183 (5)	0.084 (4)	0.027 (4)	−0.008 (3)
C20A	0.279 (14)	0.093 (5)	0.186 (9)	0.083 (7)	0.039 (9)	0.046 (6)
C20B	0.128 (12)	0.070 (9)	0.23 (2)	0.017 (7)	0.035 (12)	0.030 (10)
N1	0.0915 (16)	0.0847 (16)	0.0522 (12)	−0.0014 (13)	−0.0187 (12)	−0.0122 (11)
N2	0.0585 (11)	0.0569 (11)	0.0450 (10)	0.0036 (9)	0.0028 (8)	0.0062 (8)
O1	0.1042 (16)	0.1076 (18)	0.1084 (17)	0.0390 (14)	0.0303 (14)	−0.0284 (14)
O2	0.1248 (18)	0.0670 (13)	0.0989 (16)	0.0474 (13)	0.0125 (13)	−0.0023 (12)
O3	0.0684 (10)	0.0557 (10)	0.0694 (11)	0.0101 (8)	0.0264 (9)	0.0053 (8)
O4	0.0811 (13)	0.0715 (12)	0.0963 (14)	0.0160 (10)	0.0341 (11)	0.0318 (11)

### Geometric parameters (Å, °)

**Table:**

C1—N1	1.137 (3)	C14—H14A	0.9700
C1—C2	1.444 (3)	C14—H14B	0.9700
C2—C7	1.380 (3)	C15—H15A	0.9600
C2—C3	1.383 (3)	C15—H15B	0.9600
C3—C4	1.377 (3)	C15—H15C	0.9600
C3—H3	0.9300	C16—H16A	0.9600
C4—C5	1.380 (3)	C16—H16B	0.9600
C4—H4	0.9300	C16—H16C	0.9600
C5—C6	1.378 (3)	C17—H17A	0.9600
C5—C8	1.533 (3)	C17—H17B	0.9600
C6—C7	1.377 (3)	C17—H17C	0.9600
C6—H6	0.9300	C18—O1	1.206 (3)
C7—H7	0.9300	C18—O2	1.326 (4)
C8—C9	1.523 (3)	C19—C20A	1.370 (8)
C8—C12	1.524 (3)	C19—C20B	1.401 (9)
C8—H8	0.9800	C19—O2	1.440 (4)
C9—C10	1.346 (3)	C19—H19A	0.9700
C9—C18	1.464 (3)	C19—H19B	0.9700
C10—N2	1.377 (3)	C19—H19C	0.9700
C10—C17	1.499 (3)	C19—H19D	0.9700
C11—C12	1.349 (3)	C20A—H20A	0.9600
C11—N2	1.385 (3)	C20A—H20B	0.9600
C11—C16	1.504 (3)	C20A—H20C	0.9600
C12—C13	1.467 (3)	C20B—H20D	0.9600
C13—O4	1.201 (3)	C20B—H20E	0.9600
C13—O3	1.336 (3)	C20B—H20F	0.9600
C14—O3	1.447 (3)	N2—H2	0.8600
C14—C15	1.448 (4)		
			
N1—C1—C2	178.1 (3)	H15B—C15—H15C	109.5
C7—C2—C3	119.8 (2)	C11—C16—H16A	109.5
C7—C2—C1	120.1 (2)	C11—C16—H16B	109.5
C3—C2—C1	120.1 (2)	H16A—C16—H16B	109.5
C4—C3—C2	119.9 (2)	C11—C16—H16C	109.5
C4—C3—H3	120.0	H16A—C16—H16C	109.5
C2—C3—H3	120.0	H16B—C16—H16C	109.5
C3—C4—C5	120.9 (2)	C10—C17—H17A	109.5
C3—C4—H4	119.5	C10—C17—H17B	109.5
C5—C4—H4	119.5	H17A—C17—H17B	109.5
C6—C5—C4	118.5 (2)	C10—C17—H17C	109.5
C6—C5—C8	120.27 (18)	H17A—C17—H17C	109.5
C4—C5—C8	121.25 (19)	H17B—C17—H17C	109.5
C7—C6—C5	121.4 (2)	O1—C18—O2	120.5 (3)
C7—C6—H6	119.3	O1—C18—C9	128.2 (3)
C5—C6—H6	119.3	O2—C18—C9	111.2 (2)
C6—C7—C2	119.5 (2)	C20A—C19—C20B	74.2 (9)
C6—C7—H7	120.3	C20A—C19—O2	112.8 (5)
C2—C7—H7	120.3	C20B—C19—O2	110.7 (7)
C9—C8—C12	111.57 (17)	C20A—C19—H19A	109.0
C9—C8—C5	110.82 (16)	O2—C19—H19A	109.0
C12—C8—C5	109.63 (16)	C20A—C19—H19B	109.0
C9—C8—H8	108.2	C20B—C19—H19B	134.7
C12—C8—H8	108.2	O2—C19—H19B	109.0
C5—C8—H8	108.2	H19A—C19—H19B	107.8
C10—C9—C18	120.7 (2)	C20A—C19—H19C	132.7
C10—C9—C8	121.06 (19)	C20B—C19—H19C	109.5
C18—C9—C8	118.2 (2)	O2—C19—H19C	109.5
C9—C10—N2	119.2 (2)	H19A—C19—H19C	75.2
C9—C10—C17	127.9 (2)	C20B—C19—H19D	109.5
N2—C10—C17	112.9 (2)	O2—C19—H19D	109.5
C12—C11—N2	119.03 (18)	H19A—C19—H19D	137.2
C12—C11—C16	128.49 (19)	H19B—C19—H19D	76.0
N2—C11—C16	112.47 (18)	H19C—C19—H19D	108.1
C11—C12—C13	125.70 (19)	C19—C20A—H20A	109.5
C11—C12—C8	121.06 (18)	C19—C20A—H20B	109.5
C13—C12—C8	113.16 (18)	H20A—C20A—H20B	109.5
O4—C13—O3	121.7 (2)	C19—C20A—H20C	109.5
O4—C13—C12	123.4 (2)	H20A—C20A—H20C	109.5
O3—C13—C12	114.78 (19)	H20B—C20A—H20C	109.5
O3—C14—C15	108.1 (2)	C19—C20B—H20D	109.5
O3—C14—H14A	110.1	C19—C20B—H20E	109.5
C15—C14—H14A	110.1	H20D—C20B—H20E	109.5
O3—C14—H14B	110.1	C19—C20B—H20F	109.5
C15—C14—H14B	110.1	H20D—C20B—H20F	109.5
H14A—C14—H14B	108.4	H20E—C20B—H20F	109.5
C14—C15—H15A	109.5	C10—N2—C11	124.26 (19)
C14—C15—H15B	109.5	C10—N2—H2	117.9
H15A—C15—H15B	109.5	C11—N2—H2	117.9
C14—C15—H15C	109.5	C18—O2—C19	114.2 (3)
H15A—C15—H15C	109.5	C13—O3—C14	118.16 (19)

### Hydrogen-bond geometry (Å, °)

**Table:**

*D*—H···*A*	*D*—H	H···*A*	*D*···*A*	*D*—H···*A*
N2—H2···N1^i^	0.86	2.32	3.098 (3)	150

Symmetry codes: (i) *x*−1/2, −*y*+1/2, *z*−1/2.
